# The study of cognitive function in children with type 1 diabetes mellitus

**DOI:** 10.1186/1687-9856-2013-S1-P34

**Published:** 2013-10-03

**Authors:** NI Jia, XIN Ying

**Affiliations:** 1 Department of Pediatrics, Shengjing Hospital, China Medical University, Shenyang, Liaoning 100004, China

## Aims

To determine if frequent exposures to hypoglycemia and hyperglycemia during early childhood lead to neurocognitive deficits, and explore the possible affected factors.

## Methods

We Chosen 32 cases of children with type 1 diabetes mellitus. They were aged from 6-16 years. The duration of the disease was more than one year. We used Chinese Wechsler Intelligence Scale for measurement and analyzing cognitive function, and compared with age- and sex-matched healthy control subjects (control group). We explored the influence of glycosylated hemoglobin and hypoglycemia on cognitive function as well.

## Results

The full intelligence quotient and the verbal intelligence quotient of diabetic group were lower than the control group. In the sub-tests, as words of knowledge, category, comprehension, arithmetic, vocabulary scale points of diabetic group was significantly lower than that of control group. The verbal comprehension factor and memory/attention factor of the two groups had significantly difference. Glycosylated hemoglobin of diabetic children had a linear regression relationship with total IQ and verbal IQ.

## Conclusion

Diabetes mellitus had effects on children’s verbal intelligence quotient and attention, which affected the full intelligence quotient. Glycosylated hemoglobin was the independent risk factor to the full intelligence quotient. Larger cross-sectional and longitudinal studies of neurocognitive function are needed to define the effect of type 1 diabetes on the developing brain.

